# Does traffic exhaust contribute to the development of asthma and allergic sensitization in children: findings from recent cohort studies

**DOI:** 10.1186/1476-069X-8-17

**Published:** 2009-04-16

**Authors:** Lennart Bråbäck, Bertil Forsberg

**Affiliations:** 1 Occupational & Environmental Medicine, Department of Public Health and Clinical Medicine, Umeå University, Umeå, Sweden; 2 Department of Public Health and Research, Sundsvall Hospital, Sundsvall, Sweden

## Abstract

The aim of this review was to assess the evidence from recent prospective studies that long-term traffic pollution could contribute to the development of asthma-like symptoms and allergic sensitization in children. We have reviewed cohort studies published since 2002 and found in PubMed in Oct 2008. In all, 13 papers based on data from 9 cohorts have evaluated the relationship between traffic exposure and respiratory health. All surveys reported associations with at least some of the studied respiratory symptoms. The outcome varied, however, according to the age of the child. Nevertheless, the consistency in the results indicates that traffic exhaust contributes to the development of respiratory symptoms in healthy children. Potential effects of traffic exhaust on the development of allergic sensitization were only assessed in the four European birth cohorts. Long-term exposure to outdoor air pollutants had no association with sensitization in ten-year-old schoolchildren in Norway. In contrast, German, Dutch and Swedish preschool children had an increased risk of sensitization related to traffic exhaust despite fairly similar levels of outdoor air pollution as in Norway. Traffic-related effects on sensitization could be restricted to individuals with a specific genetic polymorphism. Assessment of gene-environment interactions on sensitization has so far only been carried out in a subgroup of the Swedish birth cohort. Further genetic association studies are required and may identify individuals vulnerable to adverse effects from traffic-related pollutants. Future studies should also evaluate effects of traffic exhaust on the development and long term outcome of different phenotypes of asthma and wheezing symptoms.

## Background

There is a strong body of evidence that traffic-related air pollutants aggravate asthmatic symptoms. Short-term exposure to air pollutants has been associated with e.g. peak flow values [1], daily symptoms [2], anti-asthmatic medication [3], emergency visits [4] and hospital admissions [5]. However, exposure to traffic exhaust may also induce onset of asthma and asthma-like symptoms in healthy individuals. Oxidative stress has been suggested as the major, underlying mechanism behind many of the toxic reactions induced by air pollutants [6]. Childhood asthma is associated with decreased levels of various components in the antioxidant defences [7]. Reactive oxygen species (ROS) and free radicals are generated by traffic related pollutants such as nitrogen dioxide (NO_2_) and particulates. Reduced glutathione, ascorbic acid, uric acid, tocopherol and other antioxidants in the airways counteract the effects of ROS. Oxidative stress arises when the neutralising antioxidant defence is overwhelmed. An inflammatory response involves an endogenous production of additional ROS and further inflammation. High levels of ROS cause a depletion of local antioxidants and lead to widespread inflammatory reactions also outside the target tissues.

There is increasing evidence mainly from experimental studies that exposure to diesel exhaust particles also has immunologic effects influencing the development of allergic sensitization. Several animal and human in vivo and in vitro studies strongly suggest that diesel exhaust particles act as adjuvants and augment the allergic reaction [8]. Combined exposure to diesel exhaust particles and antigen could switch the immune response towards IgE [9]. A recent animal study indicated that exposure to diesel exhaust particles induces epigenetic changes [10]. Concomitant exposure to ambient diesel exhaust particles and antigen in mice altered the methylation of T helper genes. Changes in the methylation affect the differentiation of the T helper cells and thereby possibly also the risk of allergic sensitization and asthma.

It has been argued that children are more susceptible to adverse effects from traffic exhaust [11]. Exposure to air pollutants early in life may have long-lasting effects [12]. The newborn child has an immature immune system and the respiratory epithelium in the developing lung has an increased permeability. A tenfold rise in the number of alveoli occurs during the first four years of life. The development of the lungs is not finished until adulthood [13]. The ratio of lung surface area to body weight is much larger in children than in adults with a huge difference in air intake per minute in relation to body weight and alveolar surface area between children and adults [14]. In addition, children tend to be more active than adults and spend more time outdoors.

Misclassification of exposure to pollutants may contribute to weak associations and inconsistent findings in population studies. In previous epidemiological research focusing on the associations between traffic pollution and asthma, allergies or sensitisation, investigators have used measured indicator pollutants as NO_2 _from stations representing the surrounding area or various surrogates to assess exposure. In the category of surrogates we find self-reported or measured traffic density in buffer zones around the home address, and self-reported or measured distance of the home to the nearest street.

More recently, exposure at the home address has also been estimated using dispersion models or land use regression models. While atmospheric dispersion models require meteorological information and emission data (i.e. emission factors for vehicle fleet), the land use regression models explain existing contrasts in monitored concentrations using, for example, road type and population density. In many cases the concentration values used come from monitoring campaigns designed to meet the needs of the modelling. A high temporal resolution is not important, but the monitoring sites must be sufficient to reflect the full range of conditions in the study population.

Also for traffic pollutants particle concentration is usually studied as particulate matter (PM) without any chemical specification. PM_10 _reflects the mass concentration of particles less than 10 microns and PM_2.5 _of particles less than 2.5 microns. Other common indicators of traffic exhaust are NO_2 _and nitrogen oxides (NO_x_), where the fraction emitted as NO_2 _tends to increase due to new diesel engines with oxidative catalysts.

Misclassification of the outcome is often a concern in questionnaire-based studies of respiratory disease, particularly in young children with less distinctive symptoms. Lower respiratory illness and wheezing symptoms are frequent in early childhood but very few of these children develop asthma. Moreover, childhood asthma is not a homogeneous disorder and different phenotypes of wheeze and asthma have been identified based on prognosis, atopic status and lung function [15]. Sensitization is assessed by objective measurements and misclassification is therefore a minor problem. Sensitization to inhalant allergens predominates after infancy [16,17]. Allergic sensitization is common in asthma and early sensitization predicts persisting symptoms [18].

A comprehensive review from WHO was presented in 2005. Rather substantial evidence suggested that the inflammatory processes associated with exposure to traffic related pollutants contributed to an increased risk of non-allergic respiratory symptoms. Antioxidants and possibly surfactant components could be important determinants of individual susceptibility [19,20]. However, it was less clear whether exposure to traffic exhaust may induce allergic sensitization. The evidence from population based studies has been weak and the findings have not been consistent as summarized in the WHO review [19,20].

Several reports based on well-designed cohort studies using different forms of exposure modelling have been published after the WHO report. The aim of this review was to assess the evidence from recent prospective studies in children to support a contribution of long-term traffic pollution to the development of asthma-like symptoms and allergic sensitization.

## Methods

We have reviewed original articles in English published since 2002 and found in PubMed in Oct 2008. We have only included cohort studies assessing potential effects of long-term exposure to traffic exhaust on respiratory health and allergic sensitization. In birth cohort studies prevalence has been seen as an equally important outcome variable as the incidence. Case-control studies and cross-sectional studies and studies on ozone have been excluded. We have also excluded studies on effects of short-term exposures.

The following search terms have been used in PubMed and Medline:

Traffic, air pollution, diesel particulate, asthma, sensitization, cohort, child

## Results

We have evaluated 15 papers based on 10 different cohort studies from Germany, the Netherlands, Sweden, Norway, the US and Japan, in all six birth cohorts and four prospective studies where the children were enrolled at or after entry at school. Most of these papers are too recent to have influenced the WHO review. The birth cohorts from Germany (Munich), the Netherlands and Sweden (Stockholm) participated in TRAPCA, an international collaboration on the impact of Traffic Related Air Pollution on Childhood Asthma [21]. Several different types of exposure variables are used: contrasts in community level of pollutants [22-24], measurements outside homes [25], distance to a large road or traffic flow [9,26,27], concentrations outside homes according to atmospheric dispersion models with emission data [28-30] and concentrations estimated with statistical models (land use regression models) including monitoring data [26,27,31-34]. Also for a specific type of exposure variable there are differences in how exposure is described, for example the averaging time used or concentration classes presented.

Data from all evaluated studies are summarized in Table 1 and 2. Studies are first by study country.

**Table 1:** Birth cohort studies

Study	Study population	Age	Exposure assessment	Agent	Range of exposure	Outcome	Relative risk	Comments
Gehring et al 2002	Birth cohort (GINI and LISA), 1756 children in the city of Munich	1–2 yrs	Individual exposure estimated from regression models Annual mean at birth	NO_2_, PM_2.5_	20–67 μg/m^3^ 12–22 μg/m^3^	Questionnaire-reported symptoms	Slightly increased OR of non-specific respiratory symptoms, significant only in males	Adjustment for important confounding variables

Morgenstern et al 2007	Birth cohort (GINA and LISA), 3577 children from the city of Munich and surrounding area	1–2 yrs	Individual exposure estimated from regression models and buffer zones variables.	NO_2_, PM_2.5_	19–72 μg/m^3^ 7–15 μg/m^3^ Annual mean at birth	Questionnaire-reported symptoms	Distance to nearest main road less than 50 m, OR 1.23 (1.00–1.51) for asthmatic bronchitis Very few children with doctor-diagnosed asthma at this age	Adjustment for important confounding variables

Morgenstern et al 2008	Birth cohort (GINA and LISA), 3066 children from the city of Munich and surrounding area	6 yrs	Individual exposure estimated from regression models and buffer zones variables	NO_2_, PM_2.5_	6–74 μg/m^3^ 19–13 μg/m^3^ Average exposure up to 6 years of age.	Questionnaire-reported symptoms Circulating IgE	Distance to nearest main road less than 50 m: OR 1.66 (1.01–2.59) for doctor-diagnosed obstructive bronchitis or asthma OR 1.30 (1.02–1.66) sensitization to pollen	Adjustment for important confounding variables. Blood samples were obtained from 1353 children (an unspecified subset) – the loss in retention rate is not commented

Brauer et al 2002	Birth cohort (PIAMA) from the Netherlands, 4,146 children at start, 3,745 at one year and 3,730 at 2 yrs.	2 yrs	Individual exposure estimated from regression models	NO_2_, PM_2.5_	13–58 μg/m^3^ 13–25 μg/m^3^ Annual mean at birth	Questionnaire-reported symptoms	Slightly but significant increased risk of upper respiratory infections	Adjustment for important confounders.

Brauer et al 2007	Birth cohort (PIAMA) 3,538 children (retention 85%) A subgroup of 713 children	4 yrs	Individual exposure estimated from regression models	NO_2_, PM_2.5_	13–58 μg/m^3^ 13–25 μg/m^3^ Annual mean at birth	Questionnaire-reported symptoms Circulating IgE	OR for IQR of PM2.5 1.32 (1.04–1.69) for doctor-diagnosed asthma ever and 1.75 (1.23–2.47) for any sensitization to food allergens	Adjustment for important confounders. High rate of retention High risk children were overrepresented in the IgE screening subgroup Low rate of sensitization to outdoor allergen

Nordling et al 2008	Birth cohort (BAMSE) of 4,089 children in Stockholm, Sweden 3,515 replied to questionnaires at 4 yrs and 2,543 delivered blood samples	4 yrs	Individual exposure based on atmospheric dispersion model, high resolution	NO_x_, Traffic PM	5–49 μg/m^3^ 1–7 μg/m^3^ (P5–P95) Annual mean at birth	Questionnaire-reported symptoms Circulating IgE	OR for 95^th ^% range of NOx 1.60 (1.09–2.36) for persistent wheeze and 1.67 (1.10–2.53) for any sensitization to pollen	Adjustment for important confounders. Analyses based on exposures during 1^st ^year of life Significant difference between extreme percentiles of exposure. Dose-response relations not presented.

Melén et al 2008	Case-cohort within the BAMSE birth cohort in Stockholm (a randomly sampled subcohort of 542 nonwheezers and 167 wheezers. In addition 375 wheezers from the original cohort)	4 yrs	Individual exposure based on atmospheric dispersion model, high resolution	NO_x_,		Questionnaire-reported symptoms Circulating IgE	Variants in the GSTP1 and TNF genes modify the association between sensitization and NOx.	

Oftedal et al 2008	Birth cohort study in Oslo, Norway 2,244 children who lived in Oslo since birth	10–11 yrs	Individual exposure based on atmospheric dispersion model with contributions from busy roads	NO_2_, PM_2.5_ PM_10_	Mean (IQR) life time estimate 29.0 (19.5) μg/m^3 ^NO2 and 12.3 (3.6) μg/m^3 ^PM2.5 Smaller ranges compared to Nordling et al	Skin prick test	No association between long-term exposure and sensitization to any allergen (except for *D. farinae*)	Very few children were sensitized to D farinae and the association with traffic exhaust was likely to be caused by confounders

Ryan et al 2005	Birth cohort study (the Cincinnati Childhood Allergy and Air Pollution Study, CCAAPS) – 622 children with at least one allergic parent were enrolled at 6 months	1 year	Individual exposure (distance to various traffic conditions) based on GIS model		Not recorded	Questionnaire-reported wheeze without a cold	Distance to stop-and-go traffic less than 100 m: OR 2.5 (1.15–5.42) for wheezing without a cold No effect from smoking	A small study with limitations in the control of confounding

Ryan et al 2007	CCAAPS See above!	1 year	Individual exposure (distance to various traffic conditions) based on GIS model and regression model estimating elemental carbon attributable to traffic	ECAT	0.30 – 0.90 μg/m^3^	Questionnaire-reported wheeze without a cold	Significant exposure-response association between ECAT level and risk of wheeze	The strength of this study is the improved exposure assessment

Clougherty et al 2007	Birth cohort – 888 pregnant women were enrolled and the caregivers of 417 children responded to questionnaires after 6–10 yrs	~7 yrs	Individual exposure based on a regression model	NO_2_	38–85 μg/m^3^	Frequent telephone or face-to-face-interviews	OR for 8 μg/m^3 ^increase in NO_2 _exposure 1.63 (1.14–2.33) for diagnosed asthma but only in children exposed to violence.	NO_2 _was included as a continuous variable. Concentration at the year of diagnosis showed the closest association. Limitations: Low retention rate, reporting bias and potential confounding

**Table 2:** Other cohort studies

Study	Study population	Age	Exposure assessment	Agent	Range of exposure	Outcome	Relative risk	Comments
Islam et al 2007	2,057 schoolchildren from 12 communities in southern California within CHS, Children's Health Study, were enrolled at 9–10 yrs of age and followed for up to 8 yrs		Community level	NO_2_, PM_2.5_ and others The levels of air pollutants were closely correlated (except for ozone)	8–75 μg/m^3^ 5–29 μg/m^3^	Incidence of doctor-diagnosed asthma Annual lung function measurements	A protective effect of a good lung function on the incidence of asthma was attenuated in communities with high levels of traffic related pollutants.	Crude exposure assessment, mainly dichotomous at community level.

Jerrett et al 2008	217 children from 11 communities in southern California within CHS were followed for eight yrs.	10 yrs at the entry of the study	2 × 2 weeks measurement outside the home of each child	NO_2_	~10 – 102 μg/m^3^	Self-reported incidence of physician diagnosed asthma	Hazard ratio was 1.29 (1.07–1.56) across the average interquartile range of ≈ 12 μg/m^3^.	Very small study size with only 26 new cases of asthma

Shima et al 2002	Prospective cohort study of 3,049 children in 8 different communities followed annually during the first six years at school	12 yrs	Community level exposure assessment.	NO_2_, PM_10_	Nearest monitoring station NO_2 _14–62 μg/m^3^ PM_10 _28–54 μg/m^3^ 10 year mean values	Annual questionnaires. Asthma defined as recurrent wheeze and medication for asthma	OR for 48 μg/m^3 ^increase of NO_2 _3.62 (1.11–11.87) for the cumulative incidence of asthma.	Limitations: There may be uncontrolled confounding at a community level. In a way, a comparison between urban and rural areas

Shima et al 2003	Prospective cohort study of 2,506 schoolchildren in 8 different communities followed annually over 4 years		Community level exposure assessment and individual exposure regarding distance between home and road.	NO_2_,	Nearest monitoring station NO_2 _14–62 μg/m^3^	Annual questionnaires Asthma defined as recurrent wheeze and medication for asthma	OR close to 4 for the cumulative incidence of asthma when children from roadside areas were compared with rural children	Crude exposure assessment. Confounders may contribute to a lower incidence of asthma in rural areas

### Germany

Three papers originated from the GINI (German Infant Intervention Programme) and LISA (Influences of Lifestyle Related Factors on the Immune System and Development of Allergies in Children) birth cohorts in Munich as a part of the TRAPCA project. The paper by Gehring et al was based on 1,756 children living in Munich City [33]. Levels of NO_2 _and PM_2.5 _at home were estimated from regression models. Annual mean values of the pollutants were related to questionnaire-reported symptoms at one and two years of age. Dry cough at night and cough without infection were associated with slightly increased odds ratios at one year but the associations were significant only in boys. The associations were attenuated at two years.

The next paper from Munich also evaluated the effects of air pollutants at one and two years but the study population was doubled since the analyses comprised all recruited children in the Munich metropolitan area (city and surroundings) [26]. The individual exposure assessment was further improved and included buffer zone variables. In contrast to the previous paper, the associations tended to be stronger in girls. Asthmatic bronchitis during the first year of life was associated with an interquartile range increase in NO_2 _whereas asthmatic bronchitis at two years was associated with a distance to main road less than 50 m, adjusted OR were 1.30 (95% CI 1.03 to 1.66) and 1.23 (95% CI 1.00 to 1.51), respectively. Very few children were reported to have doctor-diagnosed asthma.

The third paper evaluated the outcome of long-term exposure to traffic-related air pollutants at six years and encompassed the Munich metropolitan area, in all 3,066 children [27]. The individual exposure modelling was based on the home addresses at birth, two or three years and at six years. The analyses were based on current symptoms at six years. An interquartile range increase in PM_2.5 _and living less than 50 m to nearest main road were associated with an increased risk of doctor-diagnosed asthma/asthmatic bronchitis, adjusted OR 1.56 (95% CI 1.03 to 2.37) and 1.66 (95% CI 1.01 to 2.59), respectively. Distance to main road had a dose-response relationship with doctor-diagnosed asthma and hay fever with the highest prevalence rates in children living less than 50 m from the road. Determination of specific IgE antibodies was carried out in 1,353 children. Thus, sensitization was assessed in less than half of the children with available questionnaire data. An interquartile range increase in PM_2.5 _and living less than 50 m from the nearest main road were associated with an increased risk of sensitization to outdoor allergens (pollen or mould), adjusted OR were 1.52 (95% CI 1.23 to 1.87) and 1.33 (95% CI 1.00 to 1.78), respectively. Distance to a main road had a dose-response relationship with sensitization to outdoor allergens.

### The Netherlands

Two papers are based on the Dutch PIAMA (Prevention of Asthma and Mite Allergy) study as a part of the TRAPCA project. The birth cohort initially comprised 4,146 children from various communities in different parts of the Netherlands. Individual exposure to PM_2.5_, soot and NO_2 _was assessed from regression models very similar to the model used in Munich [33]. The first paper assessed the outcome at two years of age [31]. The levels of PM_2.5_, soot and NO_2 _were correlated. An interquartile increase in air pollutant concentration was associated with a slightly but significantly increased incidence of upper respiratory infections during second year of life, adjusted OR attributed to PM_2.5_was 1.14 (95% CI 1.01 to 1.42). A slightly increased risk was also observed for doctor-diagnosed asthma, although significant only for children with a diagnosis of asthma during first year of life.

The next report from the PIAMA study assessed the outcome at four years of age and data were collected from 3,538 children [35]. The individual annual average of exposure to air pollutants calculated from the address at birth was related to prevalence and cumulative incidence of reported symptoms. The odds ratios for PM_2.5_, soot and NO_2 _were fairly similar. An interquartile increase in PM_2.5 _was associated with an increased risk of wheeze ever and doctor-diagnosed asthma ever, OR 1.22 (95% CI 1.06 to 1.41) and 1.32 (95% CI 1.04 to 1.69), respectively. Analyses of specific IgE antibodies were carried out in a subgroup of 738 children where allergic mothers were over-represented. An interquartile range increase in PM_2.5 _was associated with an increased risk of sensitization to food allergens, OR 1.75 (95% CI 1.23 to 2.47). The odds ratios for sensitization were slightly reduced after sensitivity analysis comprising only children who had not moved since birth.

### Sweden

Respiratory health and allergic sensitization were assessed at four years of age amongst 3,515 children of a Swedish birth cohort (BAMSE) in Stockholm within the TRAPCA study [28,29]. Individual exposure to traffic pollutants was based on an atmospheric dispersion model with high resolution. Odds ratios for air pollutants were assessed for the difference between the fifth and the ninety-fifth percentiles within the cohort. Transient wheeze (wheeze in early life but not at four years), late-onset wheeze (wheeze only at four years) and doctor-diagnosed asthma had no association with traffic-PM_10_, traffic-NO_x _and heating SO_2 _during first year of life. In contrast, traffic-NO_x _had a significant association with persistent wheeze (wheeze both at one and four years of age), adjusted OR 1.60 (95% CI 1.09 to 2.36). The association was only significant in females. The association between traffic-NO_x _and wheeze tended to be stronger in non-atopic children. Similar but not significant associations were demonstrated also for traffic-PM_10_. Moreover, specific IgE antibodies were analyzed in 2,614 children. Sensitization to pollen was significantly associated with traffic-PM_10 _and traffic-NO_x_. Adjusted OR were 2.30 (95% CI 1.23 to 4.29) and 1.67 (1.10 to 2.53), respectively.

Another paper evaluated whether potential effects of traffic-related pollutants on doctor-diagnosed asthma, wheezing symptoms and sensitization were modified by polymorphism in genes encoding glutathione S-transferase P1 (GSTP1) and tumour necrosis factor (TNF) [28]. The study had a case-cohort design of in all 1,084 children comprising a random sample of 709 children from the cohort at four years (542 non-wheezers and 167 wheezers) and in addition, all remaining children with wheezing at four years. One of the alleles encoding GSTP1, Ala114Val, was associated with asthma, OR 2.1 (95% CI 1.4 to 3.3) and the TNF-308 allele with sensitization, OR 1.6 (95% CI 1.1 to 2.2). Polymorphism in GSTP1 gene interacted with exposure to traffic-related air pollutants. The difference between fifth and ninety-fifth percentile range of exposure to traffic-NO_x _in the cohort was associated with an increased risk of sensitization in children with the 105Val or the Ala114Val allele, adjusted OR 2.4 (95% CI 1.0 to 5.3) and 4.2 (95% CI 1.0 to 18.7), respectively. Moreover, the association between GSTP1 polymorphism and traffic-NOx on sensitization was modified by genetic variants in TNF. Thus, exposure to NO_x _was associated with an increased risk of sensitization mainly in children, who combined the TNF-308 with the GSTP1 105Val allele, adjusted OR 22.0 (95% CI 1.6 to 298).

### Norway

In all, 2,244 schoolchildren from Oslo were skin prick tested at 9–10 years of age and 24% of the children had at least one positive skin prick test [30]. All of them were living in Oslo at birth and 1,274 children were participants in an ongoing birth cohort study. Individual exposures to traffic related pollutants (NO_2_, PM_10 _and PM_2.5_) in early life and during lifetime were calculated using an atmospheric dispersion model that is adding the local contributions from busy roads. Exposure to traffic related pollutants had no association with sensitization to any allergen, any indoor or any pollen allergen. However, positive skin prick test to Dermatophagoides farinae was associated with one quartile increase of lifetime exposure to traffic pollutants. This association was no longer significant after adjustment for socioeconomic indicators suggesting that the association was caused by confounding. Similarly, sensitization to cat was associated with lifetime exposure to traffic pollutants but only in the non-cohort population[30].

### The United States

Two studies were based on children participating in the Southern California Children's Health Study (CHS). Islam et al has prospectively evaluated the incidence of asthma in 2,057 schoolchildren over a period of eight years in twelve communities [22]. Exposure to traffic related air pollutants was only assessed at area level and classified as high or low. The children were interviewed and lung function tested annually. A high lung function at the entry in the study was associated with a low risk for asthma. However, the protective effect of a high lung function was attenuated in children living in communities with a high level of pollutants.

Jerrett et al assessed onset of asthma in a sample of 217 children from 11 communities in southern California with an equal number of children from two strata in each community (above or below the median traffic exposure) [25]. At the entry of the study, residential exposure levels of NO_2 _were monitored outside the homes during two weeks in the summer and two weeks during the winter season. In all, 26 children developed asthma over a period of eight years. An interquartile range increase in residential NO_2 _was associated with an increased incidence of asthma, the hazard ratio was 1.29 (95% CI 1.07 to 1.56).

Two papers have assessed development of symptoms during the first year of life among 633 children with at least one atopic parent in an ongoing birth cohort study, the Cincinnati Childhood Allergy and Air Pollution Study (CCAAPS). In the first paper, individual exposure to traffic was assessed using a GIS model and a traffic exposure classification scheme [9]. Infants who resided less than 50 m from stop-and-go traffic had a threefold increased risk of wheeze without cold compared with those children who were unexposed. In the next paper, the individual exposure assessment also included levels of ECAT (elemental carbon attributable to traffic sources) [34]. An increasing level of ECAT was associated with an increasing risk of wheeze.

In a birth cohort from East Boston, the mothers of 413 children responded to a detailed questionnaire concerning lifetime exposure to violence [32]. Individual exposure to traffic related NO_2 _was based on a regression model. Doctor-diagnosed asthma was associated with high levels of NO_2 _at the time for the diagnosis but only in children with exposure to violence above median.

### Japan

The first paper comprised 3,049 schoolchildren from eight different urban and rural communities [24]. Respiratory symptoms were evaluated annually from the first through the sixth grades. Exposure to NO_2 _and PM_10 _were only assessed at community level from monitoring stations usually less than 1.4 km from the study schools. Incidence of asthma was associated with increasing levels of NO_2_.

In the second paper, 2,506 schoolchildren from urban and rural communities were followed for four years [23]. Exposure assessment also included the distance between residential site and the closest main road. Urban children living less than 50 m from a busy road had an increased risk of asthma or wheeze but only in comparison with rural children in the same area.

## Discussion

All epidemiological studies have certain limitations. We have in this review only included prospective studies implying that exposure to air pollutants was assessed before the development of symptoms. In all, 13 papers based on data from 9 cohorts have evaluated the relationship between traffic exposure and respiratory health. An increased risk of respiratory symptoms was demonstrated in all studies. The outcome varied, however, by the age of the child. With the limited number of studies with both the same exposure measure and the same definition of the health outcome, we found it too early to now perform a formal meta-analysis. Different types of exposure assessment have been used in these studies: traffic proximity (GIS), dispersion modelling, pollution measurements and regression models. Some studies seem to have a very high spatial resolution in exposure data, but it is difficult to compare these different approaches. However, internal validations show for example that the dispersion model in the Norwegian study [30] seems to perform less well than the model in the similar Swedish study [28,29]. Such differences may contribute to inconsistent findings.

The association between traffic-related air pollutants and respiratory health during infancy was assessed in three birth cohorts. In two of the large European birth cohorts, exposure to traffic exhaust was related to slightly increased odds ratios for coughing symptoms [33], asthmatic bronchitis [26], upper respiratory infections and doctor-diagnosed asthma [31] In the birth cohort form Cincinnati, distance to stop-and-go traffic [9] and increasing levels of traffic soot had a dose-response relationship with cough without wheeze during the first year of life [34]. Wheeze and cough induced by respiratory infections are common in early childhood. Wheezing is not specific for asthma and many of the wheezers are symptom-free when they start at school [36].

Collaborative centres in the TRAPCA study assessed potential effects of traffic exhaust also at four [29,35] or six years [27]. The findings were fairly similar. There were some inconsistencies, however. In the Dutch cohort, exposure to traffic related pollutants was associated with an increased risk of doctor-diagnosed asthma and wheeze [35]. In Munich, exposure to pollutants and distance to main road were associated with doctor-diagnosed asthma or asthmatic bronchitis whereas the association between traffic exposure and reported wheeze was less consistent [27]. In the Swedish cohort, traffic exposure had an association with persistent wheeze but no association with doctor-diagnosed asthma [28,29]. Inconsistencies in wheezing and doctor diagnosed asthma between countries could possibly be related to discrepancies in the interpretation of the term "wheeze" and variability in diagnostic routines and labelling of symptoms.

Five studies assessed the outcome in schoolchildren. In the two Japanese cohorts, urban schoolchildren exposed to traffic related pollutants and/or living close to main road had an increased prevalence of asthma in comparison with rural children [23,24]. It could not be excluded, however, that a low prevalence of asthma was attributed to protective factors related to rural living. Two cohorts were recruited from the CHS in southern California. Lung function and exposure to ambient air pollutants appeared to have interactive effects on the development of asthma in one of the studies. Exposure assessment was less sophisticated when compared with the European studies and levels of pollutants were only defined as high or low [22]. Residential exposure to NO_2 _was assessed in the other study from California but this study had limitations due to the study size and included only 26 new cases of asthma over a period of eight years. In the cohort study from East Boston, the children were followed annually from birth up to 18 years. Long-term exposure to traffic exhaust and exposure to violence appeared to have synergistic effects on the risk of asthma [32]. However, reporting of symptoms could be more likely in children exposed to violence. Control of confounding was limited also in this study due to the fairly small sample size.

Asthma is a heterogeneous disease and there are several phenotypes of childhood wheezing [37]. Potential effects of traffic related air pollutants on different wheezing phenotypes were only assessed in one of the birth cohorts [28,29]. Adjustment for relevant covariates, e.g. socioeconomic status and parental education had certain shortcomings in three of the cohorts [23,24,32]. Unmeasured residual confounding could affect the outcome in any of the studies. Nevertheless, the consistency in the results indicates that traffic exhaust contributes to the development of respiratory symptoms in healthy children. This is supported by recent genetic association studies strongly suggesting that variability in the susceptibility to air pollutants is influenced by polymorphism in genes involved in airway inflammation and oxidative stress [38,39]. Type and timing of exposures regulate the expression of the genes [40]. Glutathione-S-transferase is a catalyst of the conjugation of reduced glutathione and an important component in the protection against reactive oxygen species [41]. An interaction has been demonstrated between glutathione-S-transferase P1 (GSTP1) gene polymorphism and outdoor air pollution on asthma among schoolchildren in Taiwan [42]. Microsomal epoxide hydrolase (EPHX1) plays a role in the detoxification of polyaromatic hydrocarbons (PAHs). EPHX1 and GSTP1 variants affect the risk of asthma in children exposed to traffic exhaust [43]. Transforming growth factor (TGF)-β1 is involved in airway inflammation and the TGF-β1 gene is upregulated by oxidant stress. Living close to highways in California was associated with an increased risk of lifetime asthma in children with certain variants in the TGF-β1 gene [43].

Potential effects of traffic exhaust on the development of allergic sensitization were only assessed in the four European birth cohorts. Long-term exposure to outdoor air pollutants had no association with sensitization in ten-year-old schoolchildren in Norway [30]. In contrast, German, Dutch and Swedish preschool children had an increased risk of sensitization related to traffic exhaust despite fairly similar levels of outdoor air pollution as in Norway [27,29,35]. A strong exposure-response association was demonstrated in Germany and the Netherlands but not in Sweden, where a significant difference in sensitization was presented only for the fifth to ninety-fifth percentile difference in air pollution exposure within the cohort. Traffic related pollutants may induce the release of allergenic granules from grass pollen [44] and affect the morphology and possibly also the allergenicity of the pollen grains [45]. Exposure to traffic-related pollutants was associated with sensitization to outdoor allergens in Germany and in Sweden. In the Netherlands, however, traffic exhaust was associated with sensitization only to food allergen. There is some evidence from experimental studies in mice that exposure to diesel exhaust could counteract the development of oral tolerance against food allergens [46].

In the Norwegian study, sensitization to single allergens in subpopulations suggested that confounding and selection bias could influence the outcome. In the Netherlands, sensitisation was assessed in a subgroup of children comprising 20% of the original cohort. This sample differed in many respects from the original cohort. Vulnerability to adverse effects from environmental factors could be related to genetic polymorphism and differ between individuals or subgroups within a population. Assessment of gene-gene and gene-environment interactions on sensitization has so far only been carried out in a subgroup of the Swedish birth cohort. Exposure to traffic-related air pollutants was associated with an increased risk of sensitization at four years in children with specific variants of GSTP1 and this association was modified by polymorphism in genes encoding TNF. Such findings strongly indicate that traffic-related effects on sensitization could be restricted to individuals with a specific genetic polymorphism. Harmful effects of environmental factors are diluted and difficult to detect in a large population where only some individuals are genetically susceptible [38,47].

## Conclusion

The findings from recent cohort studies indicate that traffic exhaust contributes to the development of respiratory illness in childhood. A growing body of evidence also suggests that traffic related air pollutants may induce sensitization. So far, however, very few cohort studies have assessed effects on sensitization and the findings are not consistent. Effects on sensitization could be difficult to detect if genetic susceptibility is uncommon in the study population. Ongoing cohorts should reassess effects of traffic exhaust on respiratory health and sensitization in older children. Further genetic association studies are required and may identify individuals vulnerable to adverse effects from traffic related pollutants. Future studies should also evaluate effects of traffic exhaust on the development and long term outcome of different phenotypes of asthma and wheezing symptoms.

## Abbreviations

CCAAPS: Cincinnati Childhood Allergy and Air Pollution Study; CHS: Southern California Children's Health Study; ECAT: Elemental carbon attributable to traffic sources; EPHX1: Microsomal epoxide hydrolase; GINI: German Infant Intervention Programme; GSTP1: Glutathione S-transferase P1; LISA: Influences of Lifestyle Related Factors on the Immune System and Development of Allergies in Children; NO_x_: Nitrogen oxides; PIAMA: Prevention of Asthma and Mite Allergy; PM: Particulate matter; ROS: Reactive oxygen species; TNF: Tumour necrosis factor; TRAPCA: Traffic Related Air Pollution on Childhood Asthma.

## Competing interests

The authors declare that they have no competing interests.

## Authors' contributions

LB and BF have designed the study, reviewed the included papers and prepared the final manuscript together.
